# Elastic potentials as yield surfaces for isotropic materials

**DOI:** 10.1371/journal.pone.0275968

**Published:** 2022-10-26

**Authors:** Jorge Castro

**Affiliations:** Group of Geotechnical Engineering, Department of Ground Engineering and Materials Science, Universidad de Cantabria, Santander, Spain; National University of Ireland Galway, Galway, Ireland, IRELAND

## Abstract

This paper proposes that elastic potentials, which may be rigorously formulated using the negative Gibbs free energy or the complementary strain energy density, may be used as the yield surface of elasto-plastic constitutive models. Thus, the yield surface may be assumed in some materials as an elastic potential surface for a specific level of critical complementary strain energy density. Traditional approaches, such as the total strain energy criterion, only consider second order terms, i.e., the elastic potential is centred at the origin of the current stress state. Here, first order terms are considered, and consequently, the elastic potential may be translated, which allows to reproduce the desired level of tension-compression asymmetry. The proposed approach only adds two additional parameters, e.g., uniaxial compressive and tensile yield limits, to the elastic ones. For linear elasticity, the proposed approach provides elliptical yield surfaces and shows a correlation between the shape of the ellipse and the Poisson’s ratio, which agree with published experimental data for soils and metallic glasses. This elliptical yield surface also fits well experimental values of amorphous polymers and some rocks. Besides, the proposed approach automatically considers the influence of the intermediate stress. For non-linear elasticity, a wider range of elastic potentials, i.e., yield surfaces, are possible, such as distorted ellipsoids. For the case of incompressible non-linear materials, the yield surfaces are between von Mises and Tresca ones.

## 1. Introduction

Although the concepts of work and energy are essential in continuum solid mechanics, they are not so commonly or easily integrated in yield criteria, which limit elastic and plastic states. Notable attempts, such as the total strain energy criterion [1, 2], are not currently used. In the related field of fracture mechanics, energy is generally accepted as a criterion for crack initiation; in fact, its origin is due to Griffith [3], who originally applied the first law of thermodynamics to solve the failure problem of a cracked glass and proposed a critical energy criterion. Nowadays, the Theory of Critical Distances (e.g., [4]) allows to apply stress-based criteria, avoiding the singularity of stresses at the crack front, and, on the other hand, the average strain energy density criterion [5] is another successful method for fracture assessment. Fracture mechanics is one of the fields that the author has worked in and motives this work [6, 7], and the other is constitutive modelling of soft soils using elasto-plastic models [8, 9].

**Fig 1:**
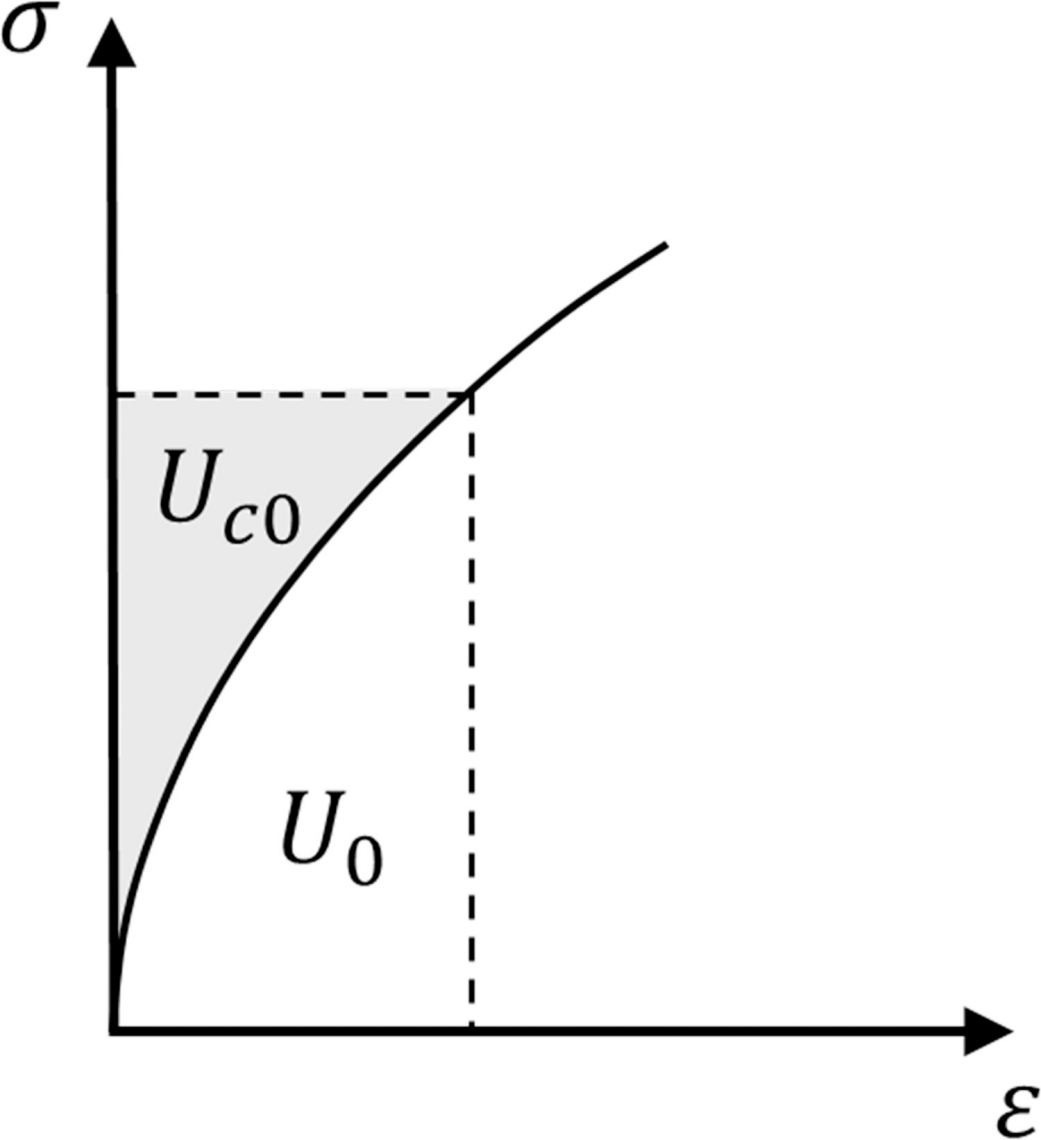
Strain energy for uniaxial stress.

Energy concepts are also helpful in providing additional techniques to solve elasticity problems (e.g., [10]). Also, a restrictive form of elasticity that is usually called Green elasticity or hyperelasticity (e.g., [11]) requires the existence of strain energy potential functions (Fig 1):

$$\sigma_{ij}=\frac{\partial U_{0}}{\partial \varepsilon_{ij}}\mathrm{and}\;\varepsilon_{ij}=\frac{\partial U_{c0}}{\partial \sigma_{ij}}$$ (1)

Cauchy stresses and small strains are considered in this paper.

Hyperelastic formulations guarantee that the first law of thermodynamics is satisfied. For isothermal conditions, the strain energy per unit volume, *U*_0_, also called strain energy density, and the complementary strain energy per unit volume, *U*_*c*0_, are equivalent to the Helmholtz free energy and Gibbs free energy with negative sign, respectively (e.g., [12]).

The search for more accurate yield surfaces has led to the development of complex surfaces with an increasing number of fitting parameters. Besides, the yield criteria are material-specific. In rocks, for example, some recent advanced models (e.g., [13]) require calibration of fitting functions, while the empirical Hoek-Brown [14] criterion is still the most popular for rocks due to its simplicity. In this manner, yield criteria should preferably be based on solid theoretical principles, cover an ample range of materials and have a few parameters that are easily calibrated, e.g., the uniaxial tension and compressive yield limits (e.g., [15]).

This paper tries to open a path for theoretically based yield surfaces using elastic potentials, which may be rigorously formulated using the negative Gibbs free energy or the complementary strain energy density (e.g., [16]). Thus, the yield surface may be assumed in some materials as an elastic potential surface for a specific level of critical complementary strain energy density. Here, rate-independent continuous materials under isothermal conditions are considered. Contrary to the total strain energy criterion, the elastic potential is not necessary centred at the origin of the current stress state because first order terms are also considered here, and consequently, the elastic potential may be translated. The proposed approach only adds two additional parameters, e.g., uniaxial compressive and tensile yield limits, to the elastic ones. This allows to correlate, for example, the shape of the yield surface and the Poisson’s ratio, which controls the shape of the elastic potential.

The basis and capabilities of the proposed approach are presented and some of its main features are validated using data available in the literature. In this manner, Section 2 presents the case of linear isotropic materials, both incompressible and compressible materials, where elastic potentials lead to von Mises and elliptical yield surfaces, respectively. Sections 3, 4, 5 and 6 present application to soils, rocks, metallic glasses and polymers, respectively. Section 7 further examines non-linear isotropic elasticity, which provides distorted elliptical yield surfaces and, for the case of an incompressible non-linear material, could lead to Tresca criterion. Finally, some conclusions are drawn.

## 2. Linear isotropic elasticity

### 2.1 Elastic potential

Linear elasticity may be easily formulated within the hyperelastic framework (e.g., [10]); it is enough to assume that the elastic potential is a quadratic form:

$$U_{c0}=U_{0}=a\sigma_{i}^{2}+b\sigma_{i}\sigma_{j}\;i,j,k=1,2,3$$ (2)

Here, contracted notation is used for the sake of brevity and elastic potentials are presented in terms of unordered principal stresses, *σ*_*i*_, for the sake of visualization in the principal stress space. As the material is isotropic, the behaviour for each principal direction should be identical. It is quickly demonstrated that:

$$\frac{{\partial^{2}U}_{c0}}{{\partial \sigma_{i}}^{2}}=2a=\frac{1}{E}\;and\;\frac{{\partial^{2}U}_{c0}}{\partial \sigma_{i}\partial \sigma_{j}}=b=-\frac{\nu }{E}$$ (3)

Thus, using the more common elastic parameters of Young’s modulus (*E*) and Poisson’s ratio (*ν*), the elastic potential for linear elasticity is:

$$U_{c0}=U_{0}=\frac{1}{2E}(\sigma_{i}^{2}-2{\nu \sigma }_{i}\sigma_{j})$$ (4)

**Fig 2:**
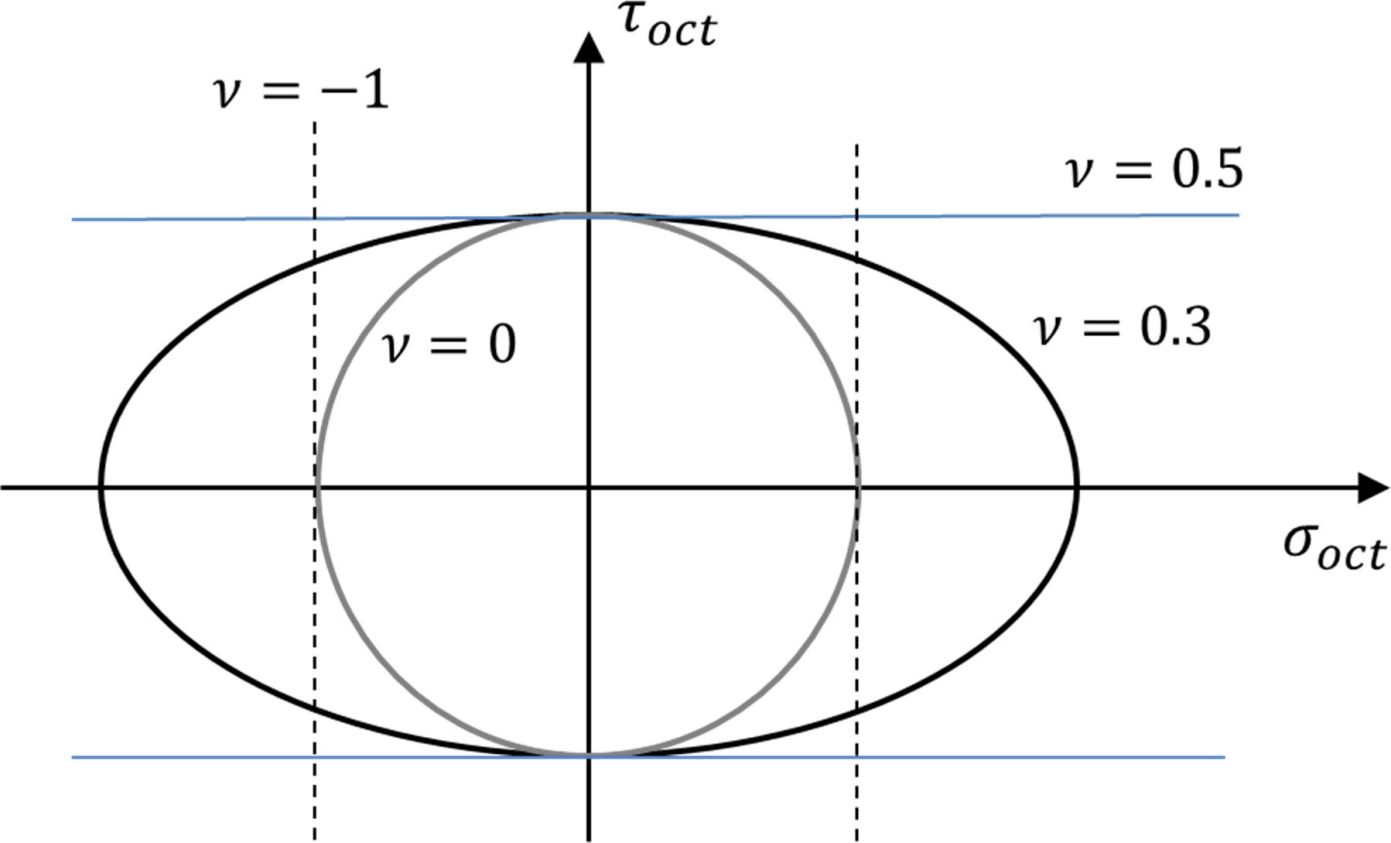
Linear elastic potentials for different Poisson’s ratios in octahedral stress plot.

The shape of the elastic potential is an ellipsoid in the principal stress space and an ellipse using the octahedral normal and shear stresses (Fig 2). For the particular case of an incompressible material (*ν* = 0.5), the elastic potential degenerates into a cylinder (Von Mises); for the case of *ν* = 0, it is a sphere, and for the strange case of *ν* = −1, it degenerates into two planar surfaces of maximum mean stress. The positive definite property of the strain energy (*U*_*c*0_≥0) gives the limit values of the Poisson’s ratio (−1≤*ν*≤1/2). From a geometrical point of view, this means that the elastic potentials should be convex surfaces (e.g., [17]).

Using Eqs (1) and (4), it may be shown that *ν* controls the strain path (shape of the elastic potential), while 1/2*E* acts like a kind of elastic multiplier:

$$\varepsilon_{ij}^{e}=\frac{\partial U_{c0}}{\partial \sigma_{ij}}=\frac{1}{2E}\frac{\partial {(2EU}_{c0})}{\partial \sigma_{ij}}$$ (5)

### 2.2 Yield surface

The proposed approach here assumes that the yield surface should correspond to an elastic potential surface for a critical value of the complementary strain energy density (*U*_*c*0_ = *U*_*c*0,*y*_). The subscript “*y*” is used here to denote the critical specific value of the strain energy that limits the elastic domain (yield surface). For linear elasticity, *U*_0_ and *U*_*c*0_ are the same and it is not necessary to distinguish. However, the critical value should be defined in terms of *U*_*c*0_ and not *U*_0_ because it is the one that constitutes a local minimum according to the principle of minimum complementary energy (e.g., [10]).


$$U_{c0,y}=U_{0,y}=\frac{1}{2E}(\sigma_{i}^{2}-2{\nu \sigma }_{i}\sigma_{j})$$ (6)

This implies a yield surface that is an ellipsoid in the principal stress space. For the particular case of an incompressible material (*ν* = 0.5), the yield surface is the von Mises cylinder (Fig 2). In this manner, the elastic potential is proposed here to be used both to establish the stress-strain relationship (Eq 4) and to define the yield surface (Eq 6).

### 2.3 Tension-compression yield asymmetry

Most materials show a tension-compression yield (and strength) asymmetry, i.e., the yield stress at compression is usually higher than that at tension. In Eq (6), no distinction between compressive and tensile stresses was made because the predicted behaviour is symmetric at tension and compression. Besides, the quadratic form proposed in Eq (2) for linear elasticity is not fully general because the linear term in stresses and the constant term, which are irrelevant for the linear elastic stress-strain behaviour, are missing. If those terms are added to Eq (2), the following general form of the complementary strain energy density is obtained:

$$U_{c0}=U_{0}=a\sigma_{i}^{2}+b\sigma_{i}\sigma_{j}+c\sigma_{i}+d$$ (7)

Introducing the linear and constant terms implies that the current (initial) state does not necessary correspond to a zero-stress and zero-strain state. This seems reasonable because the material may have previously suffered whatever strains and stresses and, from a practical point of view, only requires an incremental calculation of the elastic deformations from the current (initial) state.

In Eq (7), the strain energy density has units of energy per volume, i.e., pressure. Thus, coefficients *a* and *b* have units of the inverse of pressure (Eq 3), *c* is dimensionless and *d* has units of pressure and it may be combined with the strain energy density for the sake of simplicity. Hence, Eq (7) may be simplified by reorganizing the parameters as follows:

$$\sigma_{i}^{2}+\frac{b}{a}\sigma_{i}\sigma_{j}+\frac{c}{a}\sigma_{i}+\frac{d-U_{c0}}{a}=0$$ (8)

where *b*/*a* = −2*ν* (Eq 3). As for the Poisson’s ratio, the values of the other parameters have certain limits to ensure that *U*_*c*0_≥0.

The yield surface may be obtained by imposing a limit value of the complementary strain energy density, *U*_*c*0,*y*_ in Eq (8). Once the Poisson’s ratio is determined, the two remaining parameters may be obtained from the yield stresses for two different stress paths. For example, using the uniaxial tensile and compressive yield stresses (−*σ*_*t*_ and *σ*_*c*_), the yield surface is

$$\sigma_{i}^{2}-2\nu \sigma_{i}\sigma_{j}-(\sigma_{c}-\sigma_{t})\sigma_{i}-\sigma_{c}\sigma_{t}=0$$ (9)

or using the hydrostatic tensile and compressive yield stresses (−*p*_*t*_ and *p*_*c*_), the yield surface is

$$\sigma_{i}^{2}-2\nu \sigma_{i}\sigma_{j}-(1-2\nu )(p_{c}-p_{t})\sigma_{i}-3(1-2\nu )p_{c}p_{t}=0$$ (10)

Thus, the yield surface has 3 parameters (e.g., *ν*, *σ*_*c*_ and *σ*_*t*_, Eq 9). Please, note that compressive stresses are assumed as positive and the parameters *σ*_*t*_ and *p*_*t*_ are defined as positive (absolute) values.

The third term in Eqs (7–10) causes a translation of the elastic potential, i.e., the yield surface, which may be interpreted as a shifted origin or an initial hydrostatic stress state, *σ*_0_, so that the elastic potential ellipsoid (Fig 2) is shifted and its origin is at *σ*_0_. Please, note that *σ*_0_ is not an “apparent” or measurable initial stress and may be considered simply as a broad idealization of internal forces, stress history, atmospheric pressure… Using *σ*_0_, the elastic potential (Eq 7) or the yield surface (*U*_*c*0,*y*_) may be alternatively expressed as:

$$U_{c0}=U_{0}=\frac{1}{2E}\left({\left(\sigma_{i}-\sigma_{0}\right)}^{2}-2\nu (\sigma_{i}-\sigma_{0})(\sigma_{j}-\sigma_{0})\right)$$ (11)

The relationship between *σ*_0_ and coefficients *c* and *d* is given by Eqs (7) and (11).


$$c=-\frac{1-2\nu }{E}\sigma_{0}\;and\;d=\frac{3(1-2\nu )}{2E}\sigma_{0}^{2}$$ (12)

Using Eqs (9–11), the initial or shifting stress (*σ*_0_) may be expressed as a function of the yield stresses

$$\sigma_{0}=\frac{\sigma_{c}-\sigma_{t}}{2(1-2\nu )}$$ (13)

$$\sigma_{0}=\frac{p_{c}-p_{t}}{2}$$ (14)

## 3. Application of the linear case to soils

Soil response is clearly non-linear, but the application of the linear formulation (Section 2) gives a first approximation, as will be shown. As commonly done for soils, compressive stresses are assumed to be positive and the stress invariants *p* and *q* are used:

$$q=\sqrt{\frac{1}{2}[{\left(\sigma_{1}-\sigma_{2}\right)}^{2}+{\left(\sigma_{2}-\sigma_{3}\right)}^{2}+{\left(\sigma_{3}-\sigma_{1}\right)}^{2}]};\;p=\frac{\sigma_{1}+\sigma_{2}+\sigma_{3}}{3}$$ (15)

Thus, using those stress invariants (*p* and *q*) and *K* and *G*, the yield surface based on the elastic potential (Eq 6) may be expressed as

$$U_{c0,y}=\frac{q^{2}}{6G}+\frac{p^{2}}{2K}$$ (16)

It is necessary to apply the translation to account for the tension-compression asymmetry (Eq 11). Besides, in the case of soils, tensile stresses are usually null (*p*_*t*_≈0) and the hydrostatic compressive yielding stress (*p*_*c*_) is usually called the mean preconsolidation pressure. Hence, the translation given by Eq (14) is *p*_*c*_/2 and Eq (16) becomes

$$U_{c0,y}=\frac{q^{2}}{6G}+\frac{{\left(p-p_{c}/2\right)}^{2}}{2K}$$ (17)

The shape of the elastic potential and yield surface given by Eq (17) is completely analogous to the yield surface and plastic potential of the Modified Cam Clay (MCC) model [18]

$$f=g=q^{2}+M^{2}p(p-p_{c})=0$$ (18)

where *M* is the stress ratio at critical state and may be correlated with the critical state friction angle for triaxial compression (*σ*_2_ = *σ*_3_, ordered principal stresses) as follows

$$sin\phi_{cr}=\frac{3M}{6+M}$$ (19)

Thus, the analogy between the proposed yield surface (Eq 17) and that of the MCC model (Eq 18) gives the following equivalences:

$$M^{2}=\frac{3G}{K}=\frac{9(1-2\nu )}{2(1+\nu )}$$ (20)

$$p_{c}^{2}=8KU_{c0,y}$$ (21)

**Fig 3:**
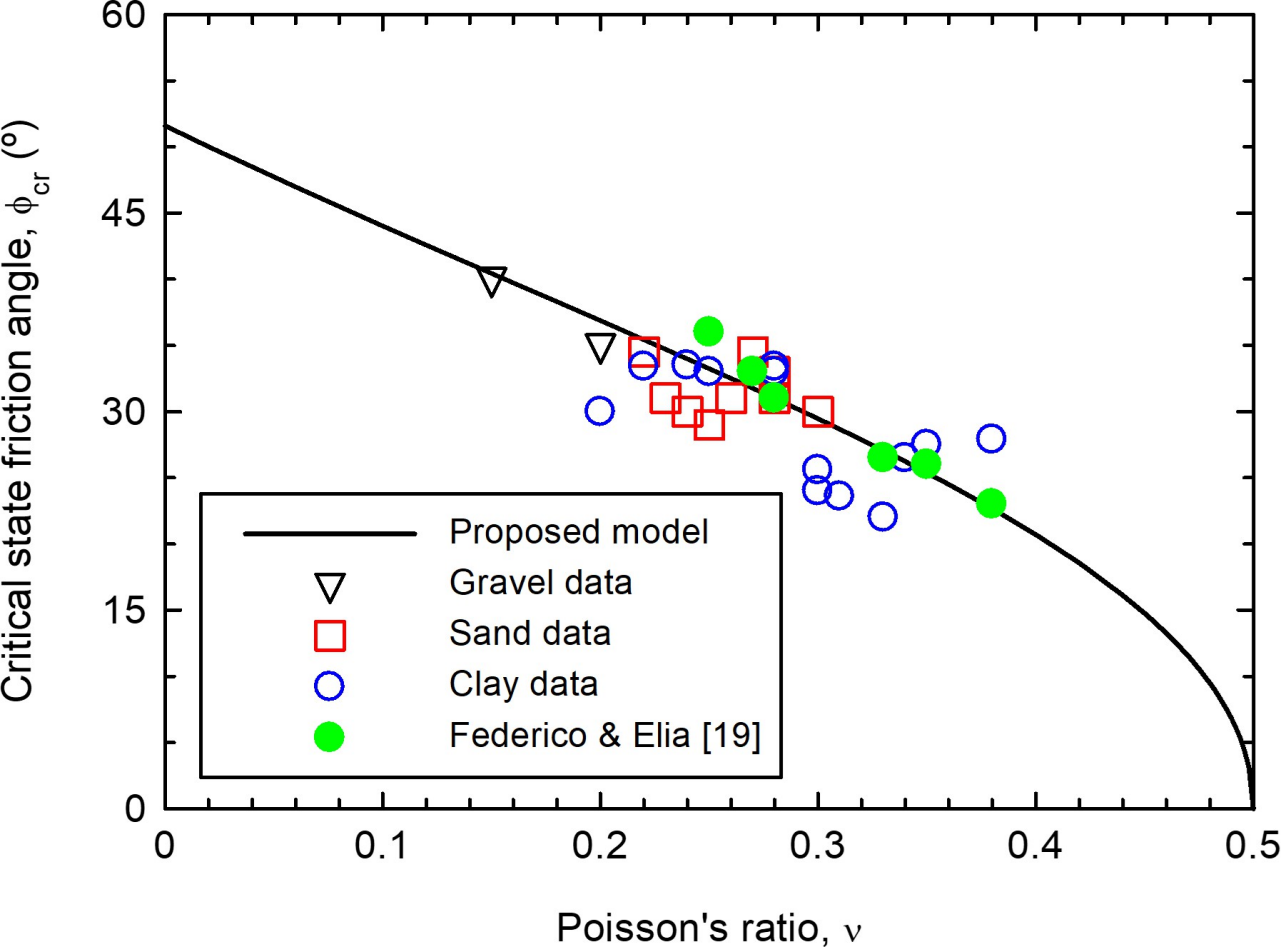
Relationship between Poisson’s ratio and critical state friction angle for soils.

Eq (20) implies a direct relationship between the Poisson’s ratio (an elastic parameter of the soil) and the stress ratio at critical state (a plastic and failure parameter of the soil). Interestingly, neither of those two parameters depend on the confining pressure. The relationship is plotted in Fig 3 and generally agrees with published values (S1 Appendix). The published values correspond to parameters for specific constitutive models calibrated from laboratory experiments, mainly drained triaxial compression tests. Some scatter in the data may arise from soil anisotropy, soil nonlinearity and Poisson’s ratio determination, calibration or specific meaning within the used constitutive model. On the other hand, Federico and Elia [19] explored several empirical correlations between the Poisson’s ratio and the friction angle of soils. For comparison with experimental data, they interpreted values provided by Wroth [20]. These data are also included in Fig 3, showing a good agreement with the proposed relationship.

## 4. Application to rocks

**Fig 4:**
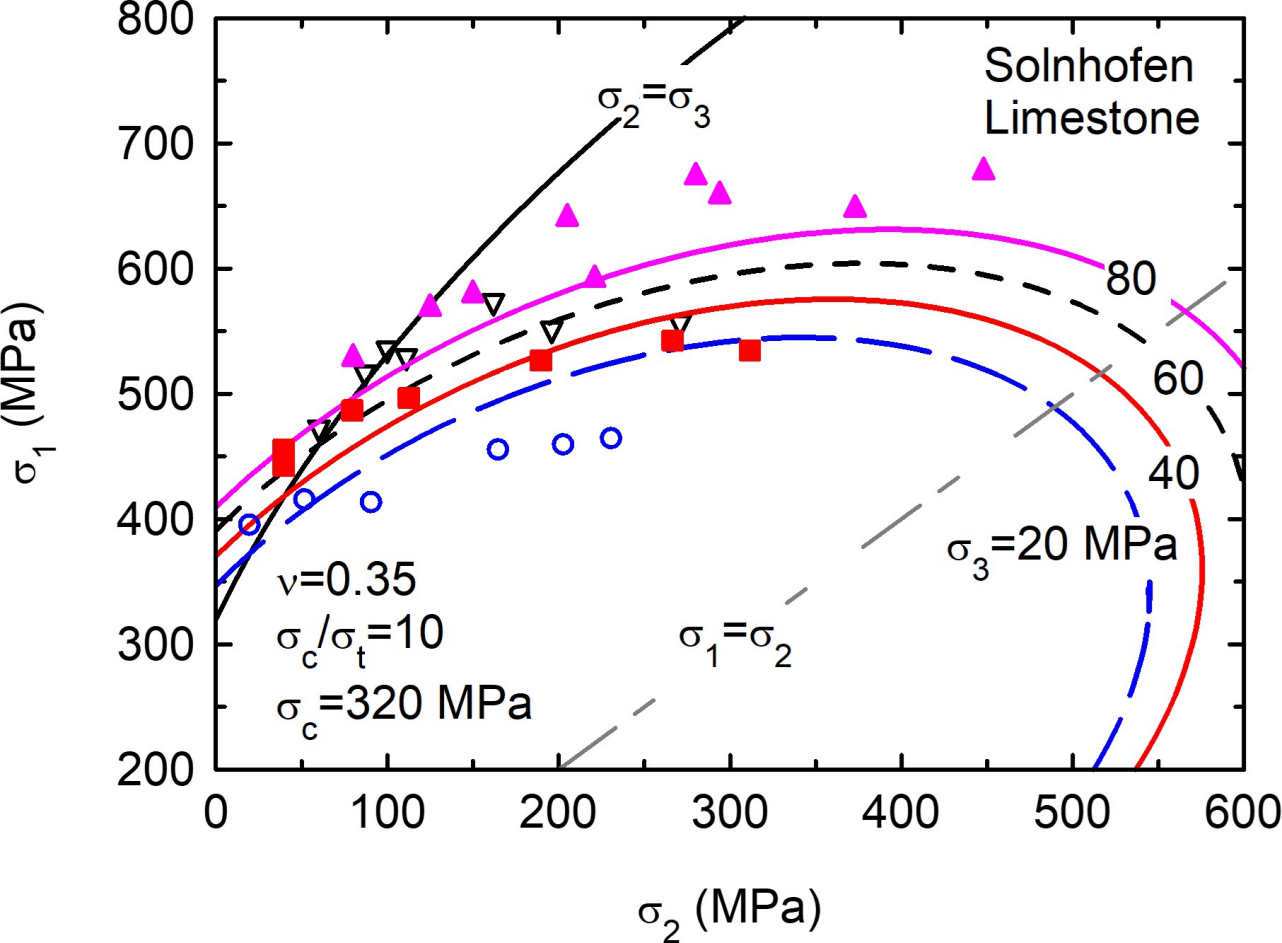
Elastic potentials as yield surfaces for Solnhofen limestone. Lines: Eq 9. Symbols: experimental data after Mogi [21]. Compressive stresses positive.

The proposed yield surface may also be applied to some rocks (Fig 4). Since most rocks behave as quasi-brittle materials, the yield surface becomes a failure envelope. The experimental data in Fig 4 correspond to Solnhofen limestone (after Mogi [21]). The main capabilities of the yield surface (Eq 9) are:

- It is able to reproduce different compression/tension strength asymmetry ratios. In fact, the ratio may directly be an input value. In rocks, which are natural materials, this is particularly useful because the uniaxial compression/tension strength ratio is usually known and it varies in the range 5–40.
- It is able to automatically consider the influence of the intermediate stress (*σ*_2_) in a logical and natural manner. Fig 4 shows experimental results of true triaxial tests [21] and the influence of *σ*_2_ is properly captured by the model.
- The shape of the failure surface (i.e., elliptical, Eq 9) is similar to a parabolic curve in the area of interest (please, refer to the black solid line, *σ*_2_ = *σ*_3_, in Fig 4). Due to the large compression/tension strength asymmetry ratio, experimental values are only available for relatively low confining stresses (area of interest). Besides, the most common failure criterion for rocks is parabolic and empirical [14].

For rock types other than Solnhofen limestone, the proposed linear model (Eq 9) usually underestimates the influence of the confining stress. Non-linear models within the proposed framework (Section 7) could be used to better reproduce the influence of the confining stress.

## 5. Application to metallic glasses

Metallic glasses are macroscopically isotropic, exhibit nearly zero tensile ductility and very limited compressive plasticity, and cannot be work-hardened. Thus, the yield surface may be assumed to be the failure surface, as previously done for rocks (Section 4). Here, the data gathered by Liu et al. [22] are reinterpreted within the proposed framework for linear elastic isotropic materials (Section 2).

For the sake of comparison with Liu et al. [22], the yield surface (Eq 11) is represented as a shifted ellipse in the Mohr’s diagram (normal and shear stresses on the failure plane (*σ*, *τ*)):

$${\left(\frac{\tau }{\tau_{y}}\right)}^{2}+{\left(\frac{\sigma -\sigma_{0}}{\sigma_{y}}\right)}^{2}=1$$ (22)

where *σ*_0_ is the initial (or shifting) stress and *τ*_*y*_ and *σ*_*y*_ are the vertical and horizontal semi-axes of the ellipse, respectively. Their ratio is the parameter that controls the shape of the ellipse, *α* = *τ*_*y*_/*σ*_*y*_, and may be related to the Poisson’s ratio, *ν*. For the sake of consistency with other parts of this paper, compressive stresses are assumed to be positive.

From Eq (11), using the Mohr’s circle and assuming triaxial (*σ*_2_ = *σ*_3_) or plane strain (*σ*_2_ = *ν*(*σ*_1_+*σ*_3_)) conditions, the following relationships between *α* and *ν* may be found:

$$\alpha^{2}=\frac{1-2\nu }{2}\;Triaxial$$ (23)

$$\alpha^{2}=\frac{1-2\nu }{2(1-\nu )}\;Plane\;strain$$ (24)

**Fig 5:**
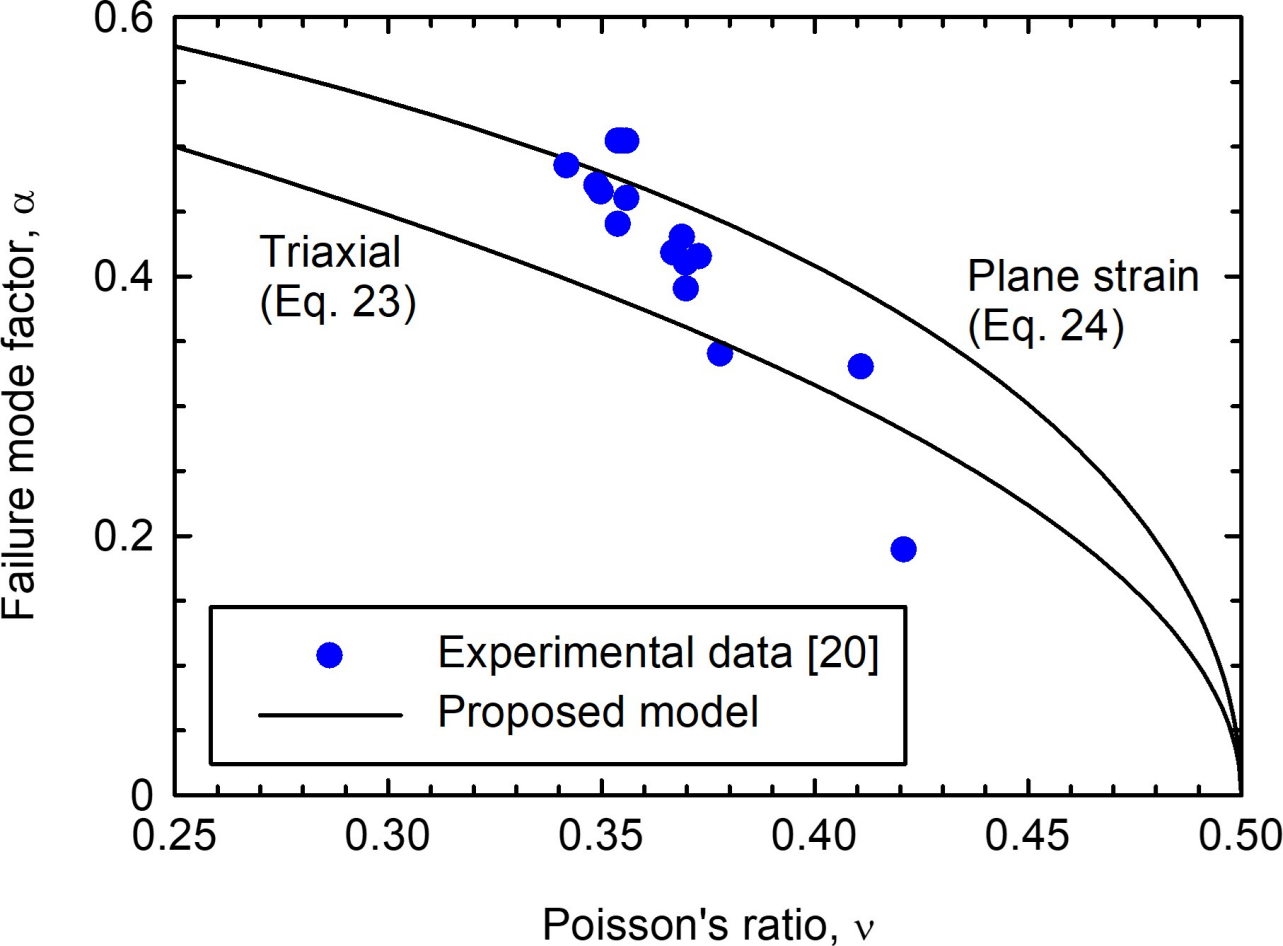
Relationship between *α* and *ν* for metallic glasses.

Fig 5 shows the relationship between *α* and *ν*. The correlation is analogous to that shown in Fig 3 for soils. Although some uncertainties arise in the comparison because the stress triaxiality of the data is not clear and experimental *α* values are influenced by their calculation process, the correlation between the shape of the yield surface and the Poisson’s ratio is clear.

Similarly to Eq (13), the shifting stress *σ*_0_ in Eq (22) may be determined based on the uniaxial compression and tensile strengths (*σ*_*c*_ and −*σ*_*t*_, respectively):

$$\sigma_{0}=\frac{\sigma_{c}-\sigma_{t}}{4\alpha^{2}}$$ (25)

**Fig 6:**
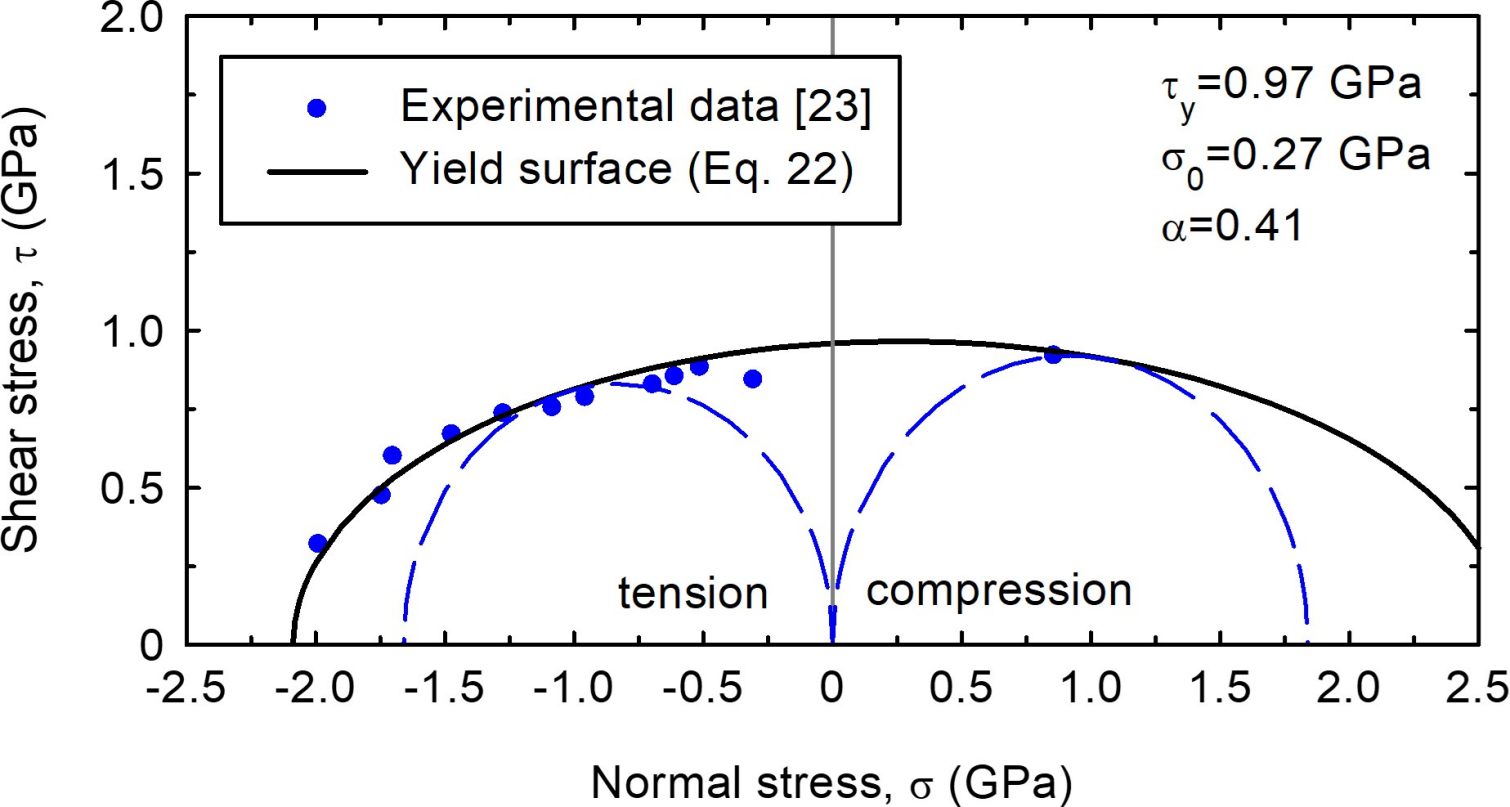
Fitted yield surface of a metallic glass tested by Qu et al. [23].

As an example of the matching properties of the proposed yield surface (Eq 22), the experimental data by Qu et al. [23] are fitted in Fig 6. The fitting is based on the tensile and compressive strengths (*σ*_*c*_ = 1.84 GPa and *σ*_*t*_ = 1.66 GPa) and the *α* value given by Qu et al. [23] (*α* = 0.41), which is in the range provided by triaxial (Eq 23, *α* = 0.36) and plane strain (Eq 24, *α* = 0.45) conditions using the reported value [22] of the Poisson’s ratio (*ν* = 0.37).

## 6. Application to amorphous polymers

**Fig 7:**
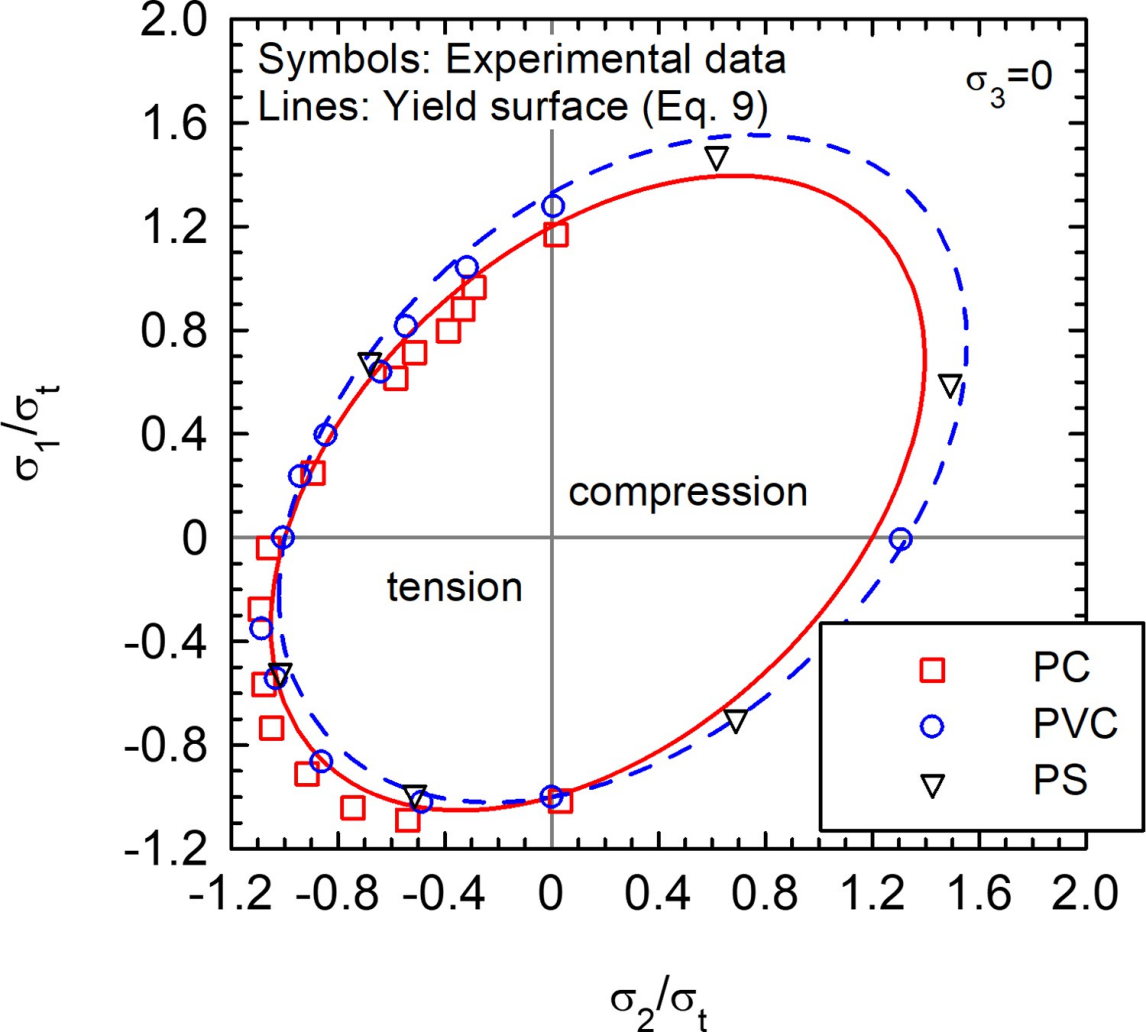
Fitted yield surfaces of amorphous polymers tested by Raghava et al. [24]. Polycarbonate (PC) (*σ*_*c*_/*σ*_*t*_ = 1.2; ν = 0.42) [24]; Polyvinyl Chloride (PVC) (*σ*_*c*_/*σ*_*t*_ = 1.33; ν = 0.38) [24]; Polystyrene (PS) [25].

Synthetic polymers are highly popular nowadays and their mechanical behavior is a subject under intense study. Raghava et al. [24] presented an interesting study on the macroscopic yield behavior of amorphous synthetic polymers. Their experimental results on polycarbonate (PC) and polyvinyl chloride (PVC) are compared with the proposed linear model (Eq 9) in Fig 7. The results are normalized by the uniaxial tensile yield stress (*σ*_*t*_). Raghava et al. [24] measured Poisson’s ratios of ν = 0.42 and 0.38 and yield asymmetry ratios of *σ*_*c*_/*σ*_*t*_ = 1.2 and 1.33 for PC and PVC, respectively.

The agreement between the experimental data and the proposed yield surface in Fig 7 is good and the slight differences between PC and PVC are also captured. To populate Fig 7 in the compressive side, the experimental data by Whitney and Andrews [25] on Polystyrene (PS) are also included. Here, the goal is just to envisage some of the potential capabilities of the proposed framework, but further and more detailed validations are required in polymers and other materials.

## 7. Non-linear isotropic elasticity

### 7.1 A general example

Some materials, such as granular materials, show a non-linear response (Fig 1), even for the elastic range. In these stress-dependent materials, the stiffness is assumed to vary with the stress state.

For isotropic materials, the complementary strain energy density may be expressed just as a function of stress invariants. The principal stresses will be here used for visualization of the elastic potential. Many different types of non-linear elasticity may be formulated within the hyperelastic framework (e.g., [12, 26]); here, for demonstration, the following complementary strain energy density function is assumed as an example:

$$U_{c0}=a\sigma_{i}^{2n}+b\sigma_{i}^{n}\sigma_{j}^{n}+c\sigma_{i}+d\;i,j,k=1,2,3$$ (26)

where *n* is a material parameter that controls the material non-linearity. This formulation has the advantage that the stiffness is stress-dependent, not just mean pressure-dependent, and it may be reduced to the linear case (Eq 7) by assuming *n* = 1. Besides, the stiffness roughly follows a power law (approximately $E_{i}\propto \sigma_{i}^{2(1-n)}$). Thus, the common range is between *n* = 1 (constant modulus) and *n* = 0.5 (roughly linear stress-dependency of the stiffness). It is worth noting that non-linear hyperelastic models always introduce a “stress-induced” anisotropy (e.g., [27]). The analysis of the non-linear elastic behaviour of this hyperelastic model is detailed in S2 Appendix.

It is convenient to introduce two mathematical tweaks in Eq (26). Firstly, negative values of the stress are not possible in Eq (26) when *n*≠1. Introducing a “back” stress, *σ*_*b*_, (pressure) is useful to avoid negative values. Hence, positive “model” stress values, σ*, are:

$$\sigma^{*}=\sigma +\sigma_{b}$$ (27)

This type of translation of the stress axes is quite common, for example, with the atmospheric pressure.

Secondly, it is useful to introduce a reference stress (pressure), $\sigma_{ref}^{*}$, so that the dimensions of constants *a* and *b* do not depend on *n* and may be expressed as a function of a reference Young’s modulus and a reference Poisson’s ratio, *E*_*ref*_ and *ν*_*ref*_, for that reference stress.

$\sigma_{ref}^{*}$ may be arbitrarily chosen, but *σ*_*b*_ is a fitting parameter that determines the stress for which the stiffness is null. Thus, Eq (26) may be expressed as:

$$U_{c0}=\frac{\sigma_{ref}^{*2}}{E_{ref}}\frac{1}{2n(2n-1)}{\left(\frac{\sigma_{i}^{*}}{\sigma_{ref}^{*}}\right)}^{2n}-\nu_{ref}\frac{\sigma_{ref}^{*2}}{E_{ref}}\frac{1}{n^{2}}{\left(\frac{\sigma_{i}^{*}}{\sigma_{ref}^{*}}\right)}^{n}{\left(\frac{\sigma_{j}^{*}}{\sigma_{ref}^{*}}\right)}^{n}+c\frac{\sigma_{i}^{*}}{\sigma_{ref}^{*}}+d$$ (28)

Similarly to the linear elastic case, the yield surface may be defined as the elastic potential (Eq 28) for a limit value of the complementary strain energy density (*U*_*c*0,*y*_). Consequently, once the non-linear elastic constants (*E*_*ref*_, *ν*_*ref*_, *n*, *σ*_*b*_) have been determined, the additional two constants of the yield surface (*c*, *U*_*c*0,*y*_−*d*) may be obtained from the yield stresses for two different stress paths, for example, the uniaxial or hydrostatic yield stresses at tension and compression.

**Fig 8:**
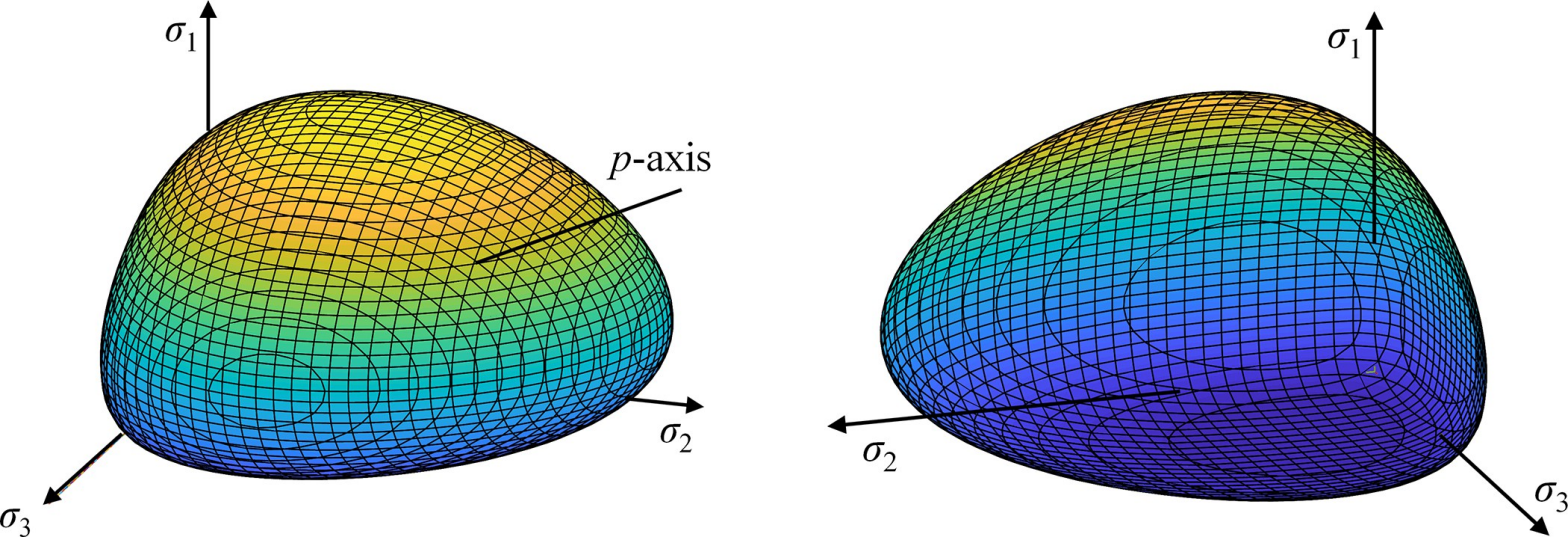
Non-linear elastic potential in 3D principal stress space (*n*≈0.5, *ν*_*ref*_ = 0.3, *p*_*t*_ = 0, *σ*_*b*_ = 0.1*p*_*c*_): (a) compressive side view; (b) tensile side view.

**Fig 9:**
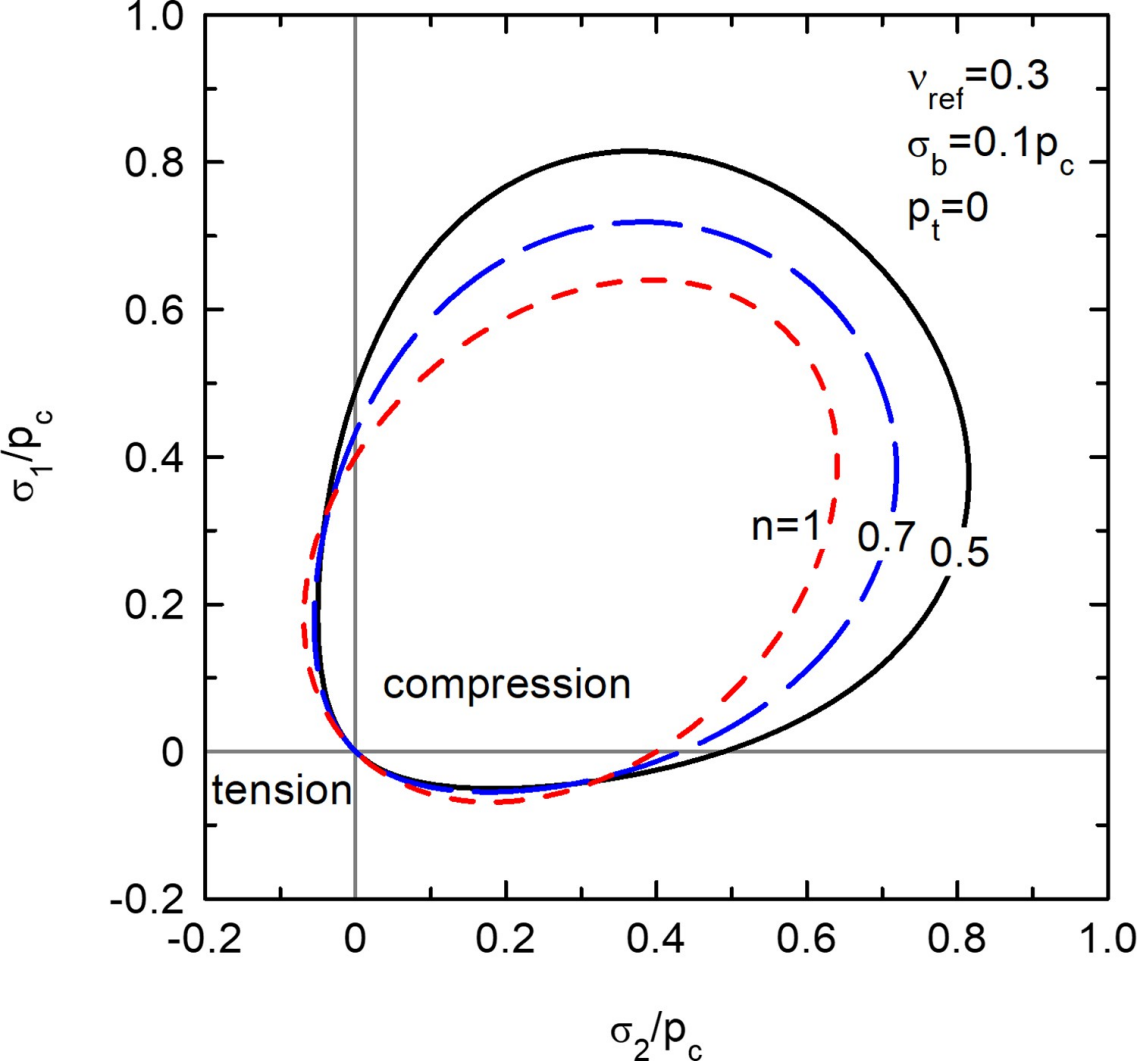
Biaxial non-linear elastic potentials (*σ*_3_ = 0).

**Fig 10:**
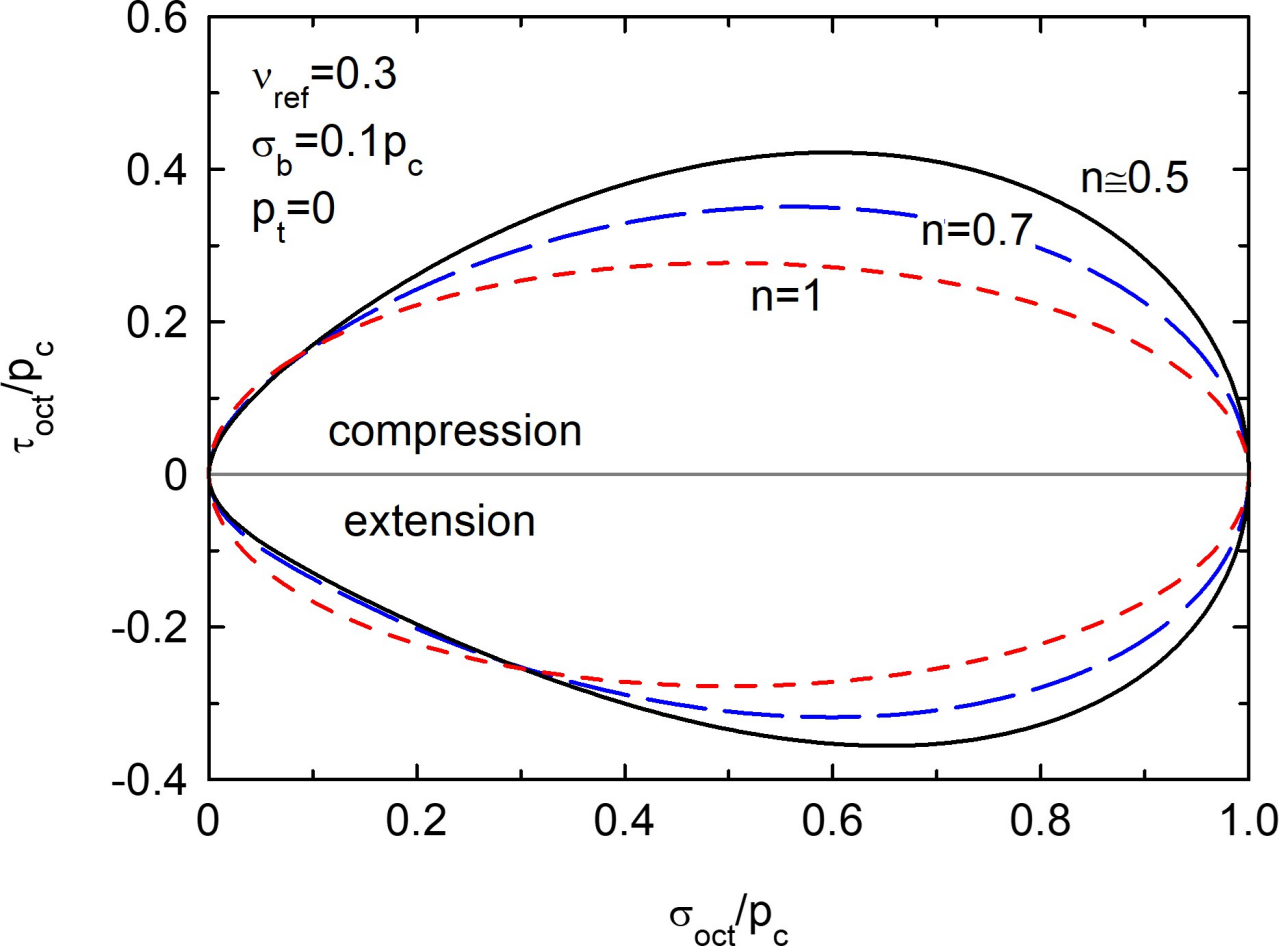
Non-linear elastic potentials in octahedral stress plot for the triaxial plane (*σ*_2_ = *σ*_3_).

**Fig 11:**
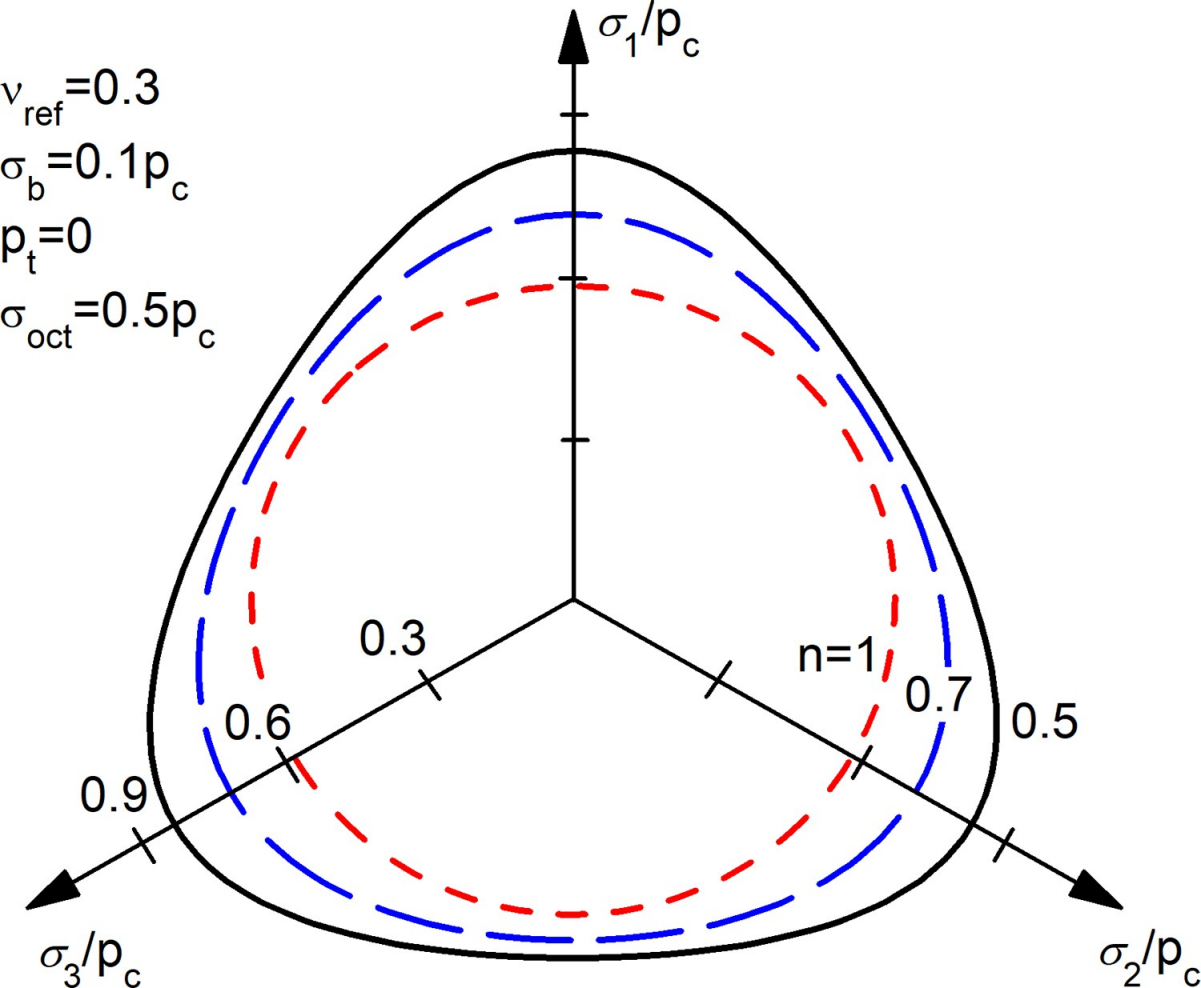
Non-linear elastic potentials in deviatoric plane.

For the non-linear case, the shape of the elastic potentials (i.e., yield surfaces) in the principal stress space are distorted ellipsoids (Figs 8–11). In Figs 8–11, simple values have been chosen for the constants, namely *p*_*t*_ = 0, *σ*_*b*_ = 0.1*p*_*c*_, *ν*_*ref*_ = 0.3 and $E_{ref}=\sigma_{ref}^{*}$ = 1 (arbitrary units). The non-linear elastic potentials reflect the asymmetries caused by the non-linear elastic behaviour, such as larger elastic regions for higher compressive stresses. This kind of distorted elliptical yield surfaces have experimentally been measured, for example, for clays (e.g., [28]). For a detailed validation of a particular model within the proposed approach, a specific laboratory campaign is required.

In the deviatoric plane (Fig 11), the stress-dependency (non-linearity) distorts the circular section for linear elastic materials (*n* = 1) towards rounded triangles, similar to Lade-Duncan [29] or Matsuoka-Nakai [30] surfaces, which in turn may be viewed as a kind of rounded Mohr-Coulomb.

### 7.2 Incompressible non-linear isotropic materials

A simple way of formulating a non-linear incompressible hyperelastic model is by using just the deviatoric component of the complementary strain energy density:

$$U_{c0}=U_{d}=\frac{1}{12G_{ref}}\left[{\left(\frac{\sigma_{1}-\sigma_{2}}{\sigma_{ref}}\right)}^{2n}+{\left(\frac{\sigma_{2}-\sigma_{3}}{\sigma_{ref}}\right)}^{2n}+{\left(\frac{\sigma_{1}-\sigma_{3}}{\sigma_{ref}}\right)}^{2n}\right]$$ (29)

Similarly to Eq (26), the quadratic power of 2 is replaced by 2*n* in Eq (29). Besides, ordered principal stresses are considered in Eq (29) to avoid negative values in the base of the 2*n* exponent. For full visualization in the principal stress space, they may be alternated. Assuming *σ*_*ref*_ = 1 (arbitrary units) for the sake of simplicity, the yield surface is:

$$12G_{ref}U_{c0,y}={\left(\sigma_{1}-\sigma_{2}\right)}^{2n}+{\left(\sigma_{2}-\sigma_{3}\right)}^{2n}+{\left(\sigma_{1}-\sigma_{3}\right)}^{2n}$$ (30)

**Fig 12:**
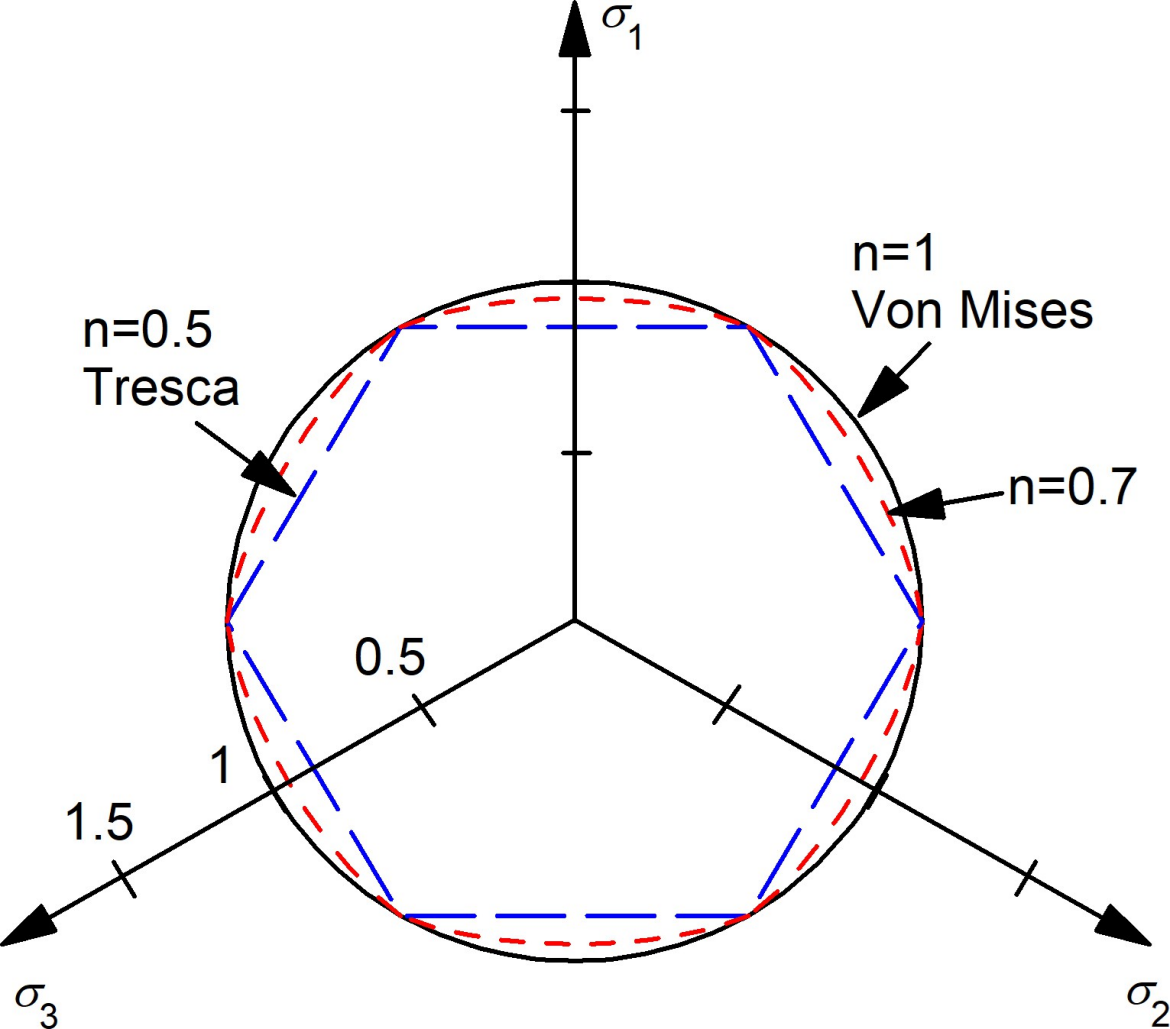
Influence of non-linearity for incompressible materials: Deviatoric plane section of the yield surface.

Eq (30) is equivalent to the Hosford yield criterion [31]. The results for several *n* values are plotted in the deviatoric or π–plane (Fig 12) and they do not vary with the mean pressure. Interestingly, the yield surface reduces to the von Mises criterion for the linear case (*n* = 1) (as already mentioned) and to the Tresca criterion for a linear stress-dependency (*n* = 0.5). The information available in the literature [32] confirms that the yielding of non-linear incompressible materials, such as soft soils under undrained conditions, is better captured by Tresca than by von Mises criterion.

## 8. Conclusions

This paper proposes that yield surfaces may be assumed to be elastic potential surfaces for specific levels of critical complementary strain energy density in some materials. Traditional approaches, such as the total strain energy criterion, only consider second order terms, i.e., the initial strain energy is null and the elastic potential is centred at the origin of the current stress state. Here, first order terms are considered, and consequently, the elastic potential may be translated, which allows to reproduce the desired level of tension-compression asymmetry.

The proposed approach shows a correlation between the shape of the yield surface and the Poisson’s ratio, which control the shape of the elastic potential. This correlation agrees well with published values in the literature for soils and metallic glasses. Besides, the yield surface is elliptical as experimentally measured. Published experimental data of amorphous synthetic polymers also agree well with this elliptical yield surface. For rocks, the tension-compression strength asymmetry is large and the elliptical yield surface in the area of interest (i.e., low confining stresses) fits well experimental data points, which have traditionally been fitted using parabolic curves. Besides, the proposed approach automatically considers, in a natural and logical manner, the influence of the intermediate stress (*σ*_2_), yet the linear case usually underestimates the influence of the confining stress and non-linear models are required.

Introducing non-linear elasticity in the proposed approach gives a wide range of elastic potentials, such as distorted ellipsoids, which are similar to those experimentally measured, for example, for clays. In the deviatoric plane, the shapes of the yield surfaces are similar to that of Matsuoka-Nakai, for example. Further experimental investigation is needed for detailed validations of specific models. For the case of incompressible non-linear materials, the yield surfaces are between von Mises and Tresca ones.

As hyperelasticity or associated plasticity, the proposed framework to derive yield surfaces using elastic potentials may be considered just as a classifying criterion and a possible approach to formulate yield surfaces in some specific materials.

Compressive stresses and strains are assumed to be positive

## Supporting information

S1 Appendix Soil data from the literature. (PDF)

S2 Appendix Example of non-linear hyperelastic model. (PDF)

## List of symbols

a,b,c,d: Parameters of the complementary strain energy function
C_ij_: Compliance matrix
E: Young’s modulus
f: Yield surface
g: Plastic potential
G: Shear modulus
K: Bulk modulus
M: Stress ratio at critical state
n: Non-linearity material parameter
p: Mean stress
p_c_: Hydrostatic compressive yield stress
p_t_: Hydrostatic tensile yield stress
q: Deviatoric stress
*U*_0_: Strain energy density
*U*_*c*0_: Complementary strain energy density
α: Shape factor in the Ellipse failure criterion
ε: Strain
ε_ij_: Strain tensor component
ν: Poisson’s ratio
σ: Normal stress
*σ**: Positive “model” stress
σ_b_: “Back” stress
σ_c_: Uniaxial compressive yield stress
σ_i_: Principal stress
σ_t_: Uniaxial tensile yield stress
*σ*_0_: Initial or shifting stress
σ_ij_: Stress tensor component
τ: Shear stress
ϕ_cr_: Critical state friction angle
**Subscripts/superscripts** 0: initial
1,2,3: principal stress directions
d,v: deviatoric, volumetric
e,p: elastic, plastic
i,j,k: principal stress directions in contracted notation
oct: octahedral
ref: reference
y: yield
