# Supplementary material for: Elastic potentials as yield surfaces for isotropic materials
Source: PLoS One. 2022 Oct 26;17(10):e0275968. doi: 10.1371/journal.pone.0275968 (PMC9604993; doi:10.1371/journal.pone.0275968)
Supplement: S1 Appendix — (PDF) [file pone.0275968.s001.pdf]

S1 Appendix. Soil data from the literature

Table S1. Published values of Poisson's ratio and critical friction angle.

Soil	*ν*	ϕ_{cr} (º)	*Reference*
Norrköping clay	0.22	33.4	Rouainia and Wood (2000)
Boston blue clay	0.24	33.5	Papadimitriou et al. (2005)
Boston blue clay	0.28	33.4	Whittle and Satabutr (2005)
Empire clay	0.31	23.6	Whittle and Satabutr (2005)
Mexico Gulf clay	0.30	25.6	Whittle and Satabutr (2005)
Lower Cromer Till	0.20	30	Papadimitriou et al. (2005)
Beaucaire Marl (reconstituted silty clay)	0.25	33.0	Masin et al. (2006)
Kaolin clay	0.34	26.5	Castro et al. (2013)
Kaolin clay	0.35	27.5	Masin (2013)
Dortmund clay	0.38	27.9	Masin (2013)
Weald clay	0.3	24	Masin (2013)
Brno clay	0.33	22	Masin (2013)
Koper clay	0.28	33	Masin (2013)
Loose fine Hostun sand	0.22	34.5	Biarez et al. (1998)
Loose fine Hostun sand	0.27	34.5	Biarez et al. (1998)
Loose fine Hostun sand	0.28	33	Biarez et al. (1998)
Loose fine Hostun sand	0.26	31	Biarez et al. (1998)
Loose fine Hostun sand	0.24	30	Biarez et al. (1998)
Loose fine Hostun sand	0.25	29	Biarez et al. (1998)
Berlin sand	0.28	31	Pestana et al. (2005)
Toyura sand	0.23	31	Pestana et al. (2005)
Toyura sand	0.3	30	Ng et al. (2015)
Latite Ballast	0.2	35	Indraratna et al. (1998)
Calcareous fine gravel	0.15	40	Castro et al. (2013)

References in Table S1

Biarez, J., Gambin, M., Gomez-Correia, A., Flavigny, E., Branque, D. 1998. Using pressuremeter to obtain parameters to elastic-plastic models for sands. In Geotechnical Site Characterization (Robertson and Mayne eds). Balkema, Rotterdam.

Castro, J., Cimentada, A., da Costa, A., Cañizal, J., Sagaseta, C. 2013. Consolidation and deformation around stone columns: Comparison of theoretical and laboratory results. Comput. Geotech. 49, 326–337.

Indraratna, B., Ionescu, D., Christie, H.D. 1998. Shear behavior of railway ballast based on large-scale triaxial tests. J. Geotech. Geoenviron. Eng. ASCE 124, 439–449.

Masin, D. 2013. Clay hypoplasticity with explicitly defined asymptotic states. Acta Geotechncia 8, 481-496.

Masin, D., Tamagnini, C., Viggiani, G., Costanzo, D. 2006. Directional response of a reconstituted fine-grained soil- Part II: Performance of different constitutive models. Int. J. Numer. Anal. Meth. Geomech. 30, 1303-1336.

Ng, C.W.W., Sun, H.S., Lei, G.H., Shi, J.W., Masin, D. 2015. Ability of three different soil constitutive models to predict a tunnel's response to basement excavation. Canadian Geotechnical Journal 52, 1685-1698.

Papadimitriou, A.G., Manzari, M.T., Dafalias, Y.F. 2005. Calibration of a simple anisotropic plasticity model for soft clays. ASCE Geotechnical Special Publication 128. Soil Constitutive Models: Evaluation, Selection, and Calibration. Geo-Frontiers Congress, 24-26 January 2005. Austin, Texas, pp. 415-424.

Pestana, J.M., Nikolinakou, M., Whittle, A.J. 2005. Selection of material parameters for sands using the MIT-S1 model. ASCE Geotechnical Special Publication 128. Soil Constitutive Models: Evaluation, Selection, and Calibration. Geo-Frontiers Congress, 24-26 January 2005. Austin, Texas, pp. 425-439.

Rouainia, M., Muir Wood, D. 2000. A kinematic hardening constitutive model for natural clays with loss of structure. Geotechnique 50, 153-164.

Whittle, A.J., Satubutr, T. 2005. Parameters for average Gulf Clay and prediction of pile set-up in the Gulf of Mexico. ASCE Geotechnical Special Publication 128. Soil Constitutive Models: Evaluation, Selection, and Calibration. Geo-Frontiers Congress, 24-26 January 2005. Austin, Texas, pp. 440-458.
