# Supplementary material for: Elastic potentials as yield surfaces for isotropic materials
Source: PLoS One. 2022 Oct 26;17(10):e0275968. doi: 10.1371/journal.pone.0275968 (PMC9604993; doi:10.1371/journal.pone.0275968)
Supplement: S2 Appendix — (PDF) [file pone.0275968.s002.pdf]

S2 Appendix. Example of non-linear hyperelastic model

This appendix presents the elastic behaviour, specifically the variation of the stiffness with the stress level, that corresponds to the complementary strain energy density function used as an example in Section 7 (Eq. 28). For simplicity, a null "back" stress is assumed ($\sigma_b = 0$), so, $\sigma^* = \sigma$. Otherwise, the corresponding isotropic stress translation must be applied.

Using equivalent moduli, the symmetric compliance matrix (C_{ij}) may be expressed as follows:

$$\begin{bmatrix} \varepsilon_1 \\ \varepsilon_2 \\ \varepsilon_3 \end{bmatrix} = \begin{bmatrix} \frac{1}{E_1} & -\frac{\nu_{12}}{E_{12}} & -\frac{\nu_{13}}{E_{13}} \\ -\frac{\nu_{12}}{E_{12}} & \frac{1}{E_2} & -\frac{\nu_{23}}{E_{23}} \\ -\frac{\nu_{13}}{E_{13}} & -\frac{\nu_{23}}{E_{23}} & \frac{1}{E_3} \end{bmatrix} \begin{bmatrix} \sigma_1 \\ \sigma_2 \\ \sigma_3 \end{bmatrix} \tag{S2.1}$$

where the equivalent moduli may be derived from the complementary strain energy density function.

$$C_{ij} = \frac{\partial^2 U_{c0}}{\partial \sigma_i \partial \sigma_j} \tag{S2.2}$$

Thus, their values are

$$C_{11} = \frac{1}{E_1} = \frac{1}{E_{ref}} \left(\frac{\sigma_1}{\sigma_{ref}} \right)^{2(n-1)} - \frac{\nu_{ref}}{E_{ref}} \frac{n-1}{n} \left(\frac{\sigma_1}{\sigma_{ref}} \right)^{n-2} \frac{\sigma_2^n + \sigma_3^n}{\sigma_{ref}^n} \tag{S2.3}$$

$$C_{12} = \frac{\nu_{12}}{E_{12}} = \frac{\nu_{ref}}{E_{ref}} \left(\frac{\sigma_1}{\sigma_{ref}} \right)^{n-1} \left(\frac{\sigma_2}{\sigma_{ref}} \right)^{n-1} \tag{S2.4}$$

Figure S2 shows the variation of the uniaxial stiffness with the corresponding principal stress (Eq. S2.3).

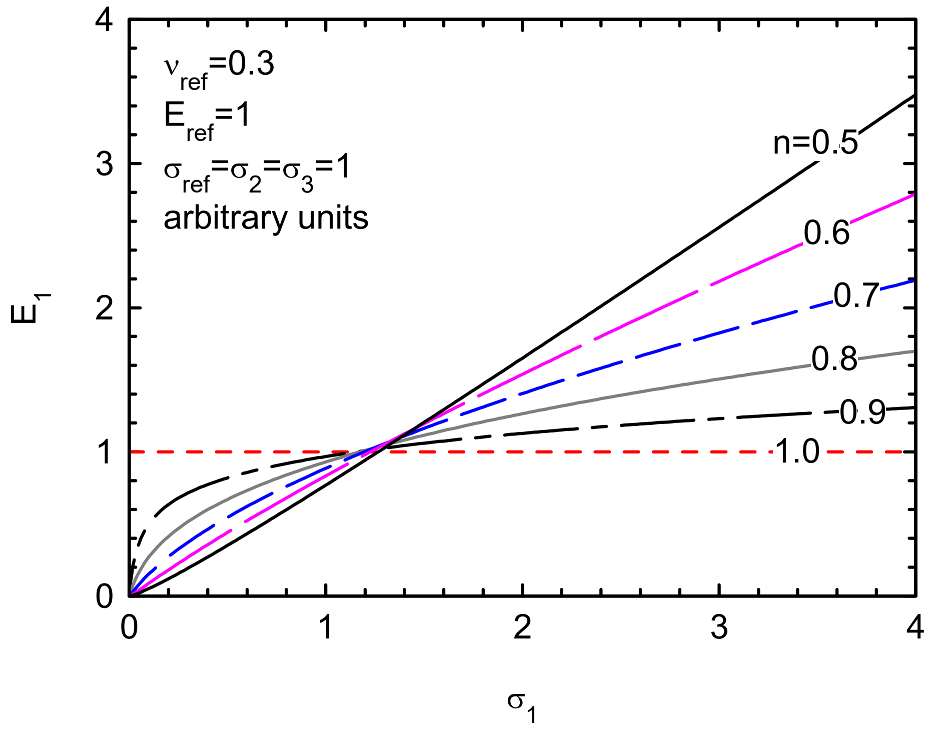

Figure S2. Stiffness variation with the stress level.
